# Underground Oxygen Deficiency Alters the Spatial Distribution Pattern of Rooting in *Alternanthera philoxeroides*

**DOI:** 10.3390/plants15142137

**Published:** 2026-07-10

**Authors:** Linsha Chen, Minjia Ge, Fusen Huang, Yingxi Xu, Mengjie Wu, Yuan Yao, Tianjiang Liu, Zirui Chen, Jiahao Luo, Xinxin Tian, Feng Lin, Bo Zeng, Xiaoping Zhang, Qiaoli Ayi

**Affiliations:** Key Laboratory of Eco-Environments in Three Gorges Reservoir Region (Ministry of Education), Chongqing Key Laboratory of Plant Ecology and Resources in Three Gorges Reservoir Region, School of Life Sciences, Southwest University, Chongqing 400715, China

**Keywords:** oxygen availability, root production, root traits, spatial distribution pattern, alligator weed

## Abstract

Terrestrial and amphibious plants frequently encounter severe oxygen deficiency in waterlogged environments. While the impacts of nutrient and water availability on root development are well documented, how underground oxygen status drives the spatial configuration of root systems remains poorly understood. In the present study, we used a custom hydroponic system to impose three dissolved oxygen treatments corresponding to 5%, 50%, and 100% air saturation and investigated the spatial rooting responses of *Alternanthera philoxeroides*. Our results demonstrated that low oxygen levels significantly restricted overall plant growth, reducing total root number, total length, and biomass allocation to the root system. However, under hypoxic conditions, plants actively altered their spatial rooting patterns by producing more roots at the top solution layers and fewer roots in the bottom layers, a spatial stratification that was more pronounced under 5% than 50% air saturation. Specifically, low dissolved oxygen availability was associated with an asymmetrical distribution of root traits, with greater root number, length, surface area, volume, and fork number on nodes closer to the solution surface than on deeper nodes. Conversely, well-oxygenated conditions (100% air saturation) promoted a more uniform root trait distribution across all vertical layers. These findings reveal a dissolved oxygen-related spatial rooting pattern, demonstrating that *A. philoxeroides* actively modulates its root architectural traits as a primary strategy to maximize oxygen acquisition in hypoxic environments, rather than relying solely on physiological tolerance.

## 1. Introduction

Resource availability and environmental disturbances are the major factors driving plant functioning and survival strategies [1]. The root system is of fundamental importance for plants to navigate these factors, as it anchors the plant to substrates and actively takes up water and nutrients [2,3]. Because root growth and resource uptake are active, energy-dependent processes regulated by ATP production, ambient oxygen is a fundamentally critical resource for root development [4,5,6]. When terrestrial or wetland plants encounter underground oxygen deficiency due to soil compaction or flooding, this metabolic process is severely compromised [7,8]. Historically, research on plant responses to such hypoxic stresses has overwhelmingly focused on internal anatomical adaptations [9,10], such as the formation of aerenchyma, to facilitate internal gas transport or barriers to radial oxygen loss (ROL) [11,12]. However, while these deep internal physiological mechanisms are well-documented, they represent only a fraction of the plant’s survival strategy. The fundamental macroscopic ecological mechanism of root architectural plasticity in response to external oxygen gradients remains largely unaddressed [13,14].

The necessity for investigating this root architectural plasticity is dictated by severe biophysical constraints in subterranean environments [15]. Atmospheric oxygen transports into media primarily by diffusion, a process that is slower in water than in air by a factor of 10^4^ [16,17]. According to diffusion laws, this physical limitation inherently creates a strict vertical oxygen gradient in flooded or compacted soils: oxygen content is highest at the atmosphere–medium interface and gradually decreases along the downward diffusion path [18]. Under such heterogeneous conditions, deep underground plant parts cannot acquire sufficient oxygen, which inevitably hampers localized ATP production and deep root construction [19,20]. This sharp vertical oxygen gradient therefore acts as a potent environmental driver that triggers root architectural plasticity [21]. To optimize resource acquisition and manage systemic energy deficits across these heterogeneous layers, plants must dynamically reconfigure their root branching, length, and spatial placement in direct response to local oxygen availability [22]. For example, in highly adaptable amphibious species such as *Rumex palustris* and *Solanum dulcamara*, severe subterranean hypoxia actively suppresses the downward growth of the primary root system to prevent futile energy investment; simultaneously, it triggers the rapid emergence of a dense adventitious root network at the well-oxygenated atmosphere–water interface to maximize systemic oxygen acquisition [23,24]. Consequently, we hypothesize that plants do not merely rely on internal aeration to survive; instead, they exhibit robust architectural plasticity by altering their macroscopic spatial rooting configuration, selectively proliferating more roots in shallow, relatively oxygenated layers while restricting deep root formation [25]. Yet, empirical evidence testing this spatial reallocation under controlled dissolved oxygen conditions remains limited. Furthermore, documenting this spatial shift is ecologically significant, as relying on shallow root systems to cope with underground hypoxia may introduce a critical survival trade-off, potentially weakening the plant’s mechanical stability and increasing its susceptibility to uprooting by gusty winds or strong flood currents [26,27].

To disentangle this spatial dynamic, we aimed to determine whether external oxygen gradients serve as a primary environmental driver for root architectural plasticity and to quantify the spatial reallocation of roots as a functional trade-off. *Alternanthera philoxeroides* (Mart.) Griseb. (*A. philoxeroides*) is a highly invasive amphibious species globally recognized for its exceptional submergence tolerance [28,29]. Upon submergence, it exhibits extreme morphological plasticity, rapidly developing adventitious roots on submerged nodes [30,31]. This inherent ability to continuously produce roots along a vertical stem axis makes it an ideal model organism to decouple general growth inhibition from active spatial root reconfiguration driven by oxygen availability [32,33]. By subjecting *A. philoxeroides* to controlled dissolved oxygen treatments in a hydroponic system, we evaluated plant growth, fresh-mass allocation, root architectural traits, and, most importantly, the spatial distribution pattern of rooting. This study aims to fill the knowledge gap regarding macroscopic spatial rooting strategies, providing essential insights into how plants dynamically exploit vertical space to mitigate underground oxygen deficiency.

Here, we hypothesize that under severe underground hypoxia, plants exhibit robust architectural plasticity by restricting deep root formation, while selectively proliferating adventitious roots in shallow, oxygenated layers to maximize systemic oxygen acquisition. By subjecting *A. philoxeroides* to controlled dissolved oxygen treatments in a hydroponic system, we assessed plant growth, fresh-mass allocation, and root architectural traits such as the surface area-to-volume ratio (SA: V). More importantly, we stress-tested the hypothesis by quantifying the spatial distribution pattern of rooting. This study expands the current paradigm of hypoxia tolerance by demonstrating how spatial rooting strategies serve as a critical mechanism for plant resilience in fluctuating aquatic environments.

## 2. Results

### 2.1. Plant Growth and Biomass Allocation

**Figure 1:**
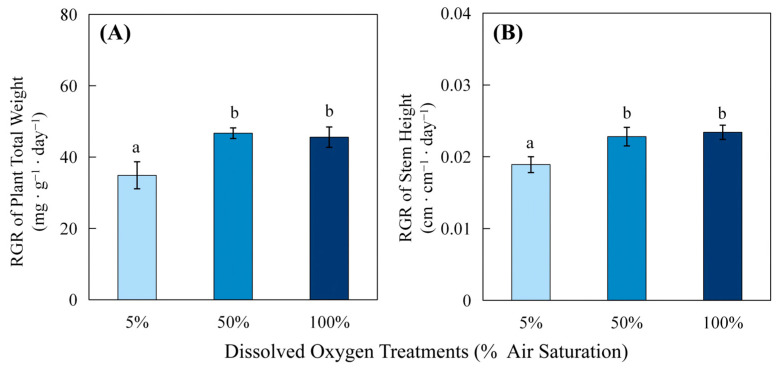
Relative growth rates of *A. philoxeroides* in terms of plant fresh weight (RGR_FW_) (**A**) and stem height (RGR_H_) (**B**) under three dissolved oxygen treatments corresponding to 5%, 50%, and 100% air saturation in Hoagland’s solution at 100% strength (means ± s.e., *n* = 10). Different lowercase letters indicate significant differences among treatments according to Duncan’s multiple range test (*p* < 0.05).

**Figure 2:**
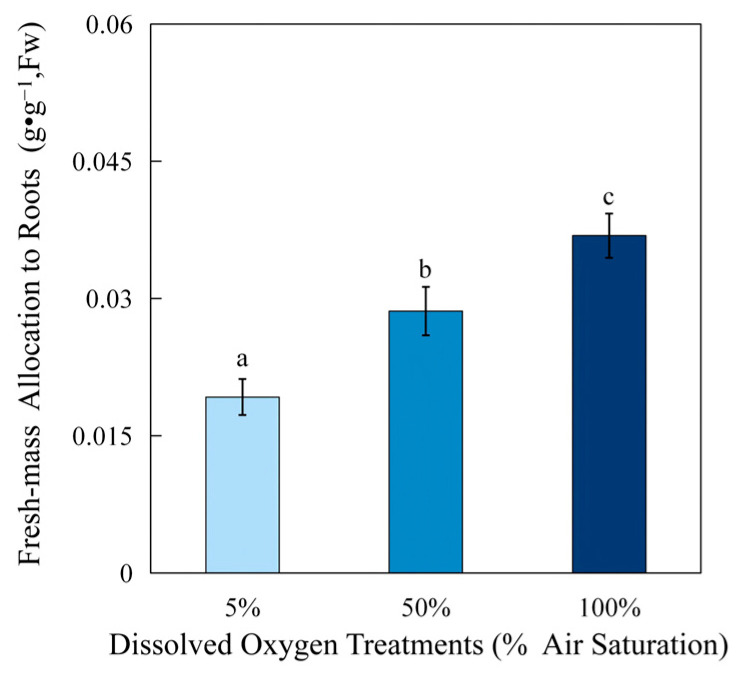
Fresh-mass allocation to roots in *A. philoxeroides* plants under three dissolved oxygen treatments corresponding to 5%, 50%, and 100% air saturation in Hoagland’s solution at 100% strength (means ± s.e., *n* = 10). Different lowercase letters indicate significant differences among treatments according to Duncan’s multiple range test (*p* < 0.05).

The growth of *A. philoxeroides* was highly correlated with the available underwater (underground) oxygen content (Figure S1). The plants grown in solution with 5% air saturation (pO_2_ 1.04 ± 0.029 kPa) had lower relative growth rates of fresh weight (RGR_FW_) and stem height (RGR_H_) than those of plants grown in solutions with 50% and 100% air saturation (pO_2_ 10.2 ± 0.038 kPa and 20.6 ± 0.102 kPa, respectively). The plants grown in solutions with 50% and 100% air saturation did not show significant differences in RGR_FW_ and RGR_H_ (Figure 1). In all plants, the fresh-mass allocation to roots increased significantly with increasing dissolved oxygen concentration in water; the plants grown in the solution with 100% air saturation had the highest biomass allocation to roots (Figure 2).

### 2.2. Root Architectural Traits

**Figure 3:**
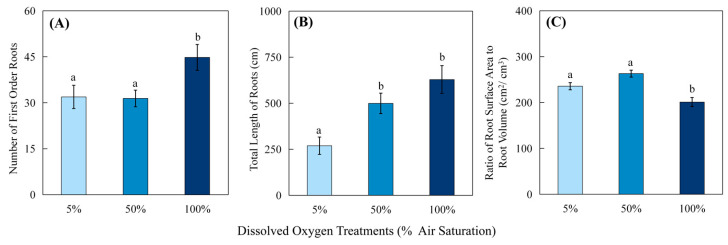
Root number (**A**), total root length (**B**), and surface area-to-volume ratio (SA: V) (**C**) of *A. philoxeroides* plants under three dissolved oxygen treatments corresponding to 5%, 50%, and 100% air saturation in Hoagland’s solution at 100% strength (means ± s.e., *n* = 10). Different lowercase letters indicate significant differences among treatments according to Duncan’s multiple range test (*p* < 0.05).

Dissolved oxygen concentration in water affected the root production and root architecture of partially submerged *A. philoxeroides* plants (Figure 3). Dissolved oxygen shortage in water reduced the number of roots produced by the treated plants (Figure 3A). Moreover, the plants grown in the solutions with 5% air saturation had the shortest total root length, whereas the plants grown in the solutions with 100% air saturation had the longest total root length (Figure 3B). Additionally, the roots became more slender under 5% air saturation treatment, as indicated by their increased surface area-to-volume ratio, whereas the roots produced by the plants treated with 100% air saturation had the lowest ratio of root surface area to root volume (Figure 3C).

### 2.3. Spatial Distribution Pattern of Roots

**Figure 4:**
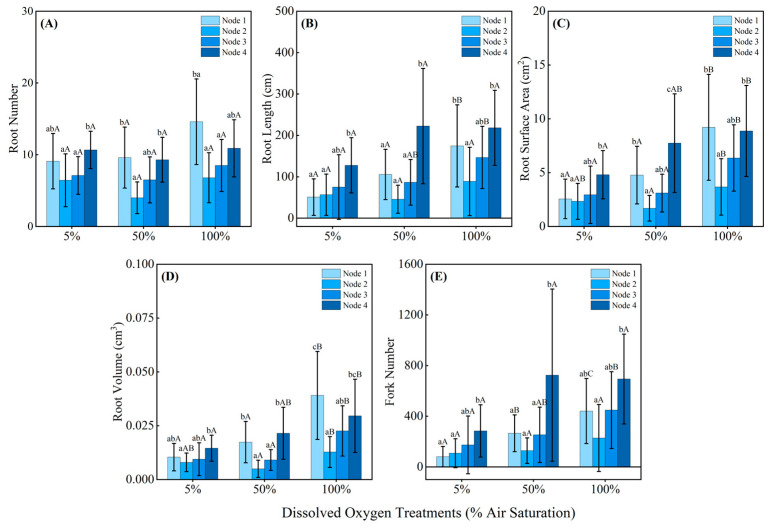
Spatial distribution of adventitious root traits among submerged stem nodes of *A. philoxeroides* under different dissolved oxygen treatments. Root traits were measured separately for the 1st, 2nd, 3rd, and 4th submerged stem nodes, with the 1st node being furthest from the water surface and the 4th node being nearest to the water surface. Panels show (**A**) root number, (**B**) root length, (**C**) root surface area, (**D**) root volume, and (**E**) root fork number (means ± s.e., *n* = 10). Different lowercase letters indicate significant differences among nodes within the same dissolved oxygen treatment, and different uppercase letters indicate significant differences among dissolved oxygen treatments for the same node, according to Duncan’s multiple range test (*p* < 0.05).

All nodes submerged in the solutions produced roots; however, under different air-saturation treatments, the plants exhibited different spatial patterns of root formation (Figure 4; Figure S2). Under 100% air saturation, root traits were more evenly distributed among submerged nodes. For root number and root volume, the 1st node, which was furthest from the water surface, generally showed higher values than the 4th node, which was nearest to the water surface. In contrast, under reduced oxygen availability, especially under 5% air saturation, root formation tended to be concentrated in the upper submerged nodes. The 4th node showed relatively higher root number, root length, root surface area, root volume, and root fork number than the lower submerged nodes, indicating an upward shift in root development under oxygen deficiency (Figure 4).

**Figure 5:**
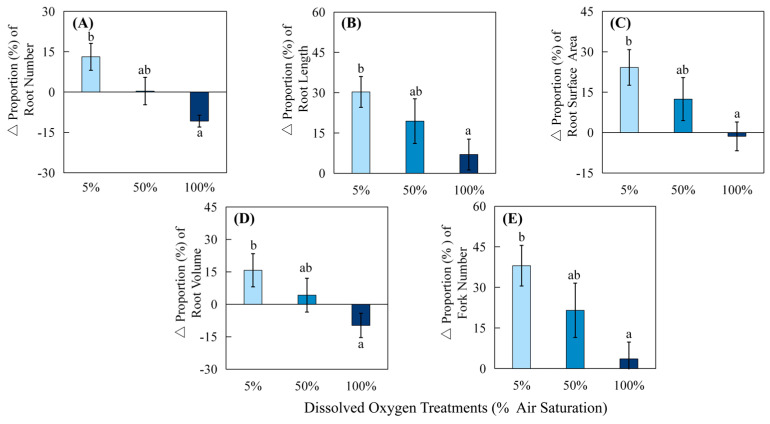
Proportion difference (Δ Proportion) (%) in root number (**A**), root length (**B**), root surface area (**C**), root volume (**D**), and root fork number (**E**) between the 4th underwater node (the nearest node to water surface) and the 1st underwater node (the furthest node to water surface) in *A. philoxeroides* plants under three dissolved oxygen treatments corresponding to 5%, 50%, and 100% air saturation in Hoagland’s solution at 100% strength (means ± s.e., *n* = 10). The proportions (%) of root number, root length, root surface area, root volume, and root fork number on the 4th (and the 1st) node to all nodes were calculated to explore Δ Proportion. Different lowercase letters indicate significant differences among treatments according to Duncan’s multiple range test (*p* < 0.05).

To further quantify this shift in rooting position, the proportional difference between the 4th and 1st submerged nodes was calculated for each root trait (Figure 5). When plants were grown in solution with 5% air saturation, they produced more roots on the nodes nearer to the water surface than on nodes further from the water surface, which was clearly shown by an increased positive proportion difference (Δ proportion) between the 4th node (the nearest to the water surface) and the 1st node (the furthest from the water surface) in root number, root length, root surface area, root volume, and root fork number (Figure 5). Comparatively, when plants were grown in solution with 100% air saturation, the Δ proportion values of root number and root volume between the 4th and 1st node were negative, which indicates that the roots produced on the 4th node had smaller total number and volume than those of roots produced on the 1st node; the Δ proportion values of root length, root surface area, and root fork number were positive, but smaller than those of plants in 5% and 50% air-saturation treatments (Figure 5).

Overall, the results from both the node-specific root trait distribution and the Δ proportion analysis showed that underwater oxygen deficiency altered the spatial distribution pattern of adventitious rooting in *A. philoxeroides*. With decreasing dissolved oxygen availability (from 100%, 50% to 5% air saturation), plants tended to form more roots with larger length, surface area, volume, and higher ramification on nodes nearer the water surface (which was in close contact with atmosphere) than on nodes further from the water surface.

## 3. Discussion

### 3.1. Plant Growth, Energy Trade-Offs, and Root Architectural Plasticity

There are notable examples of differential architectural or physiological responses of plants to deficiencies in different resources [34]. Among these responses, the most important ones are those related to increases in resource uptake and efficient utilization of limited resources [35,36]. Our comparative study of the responses of terrestrial plant *A. philoxeroides* to oxygen deficiency adds a novel contribution to this topic.

In the present study, we found that oxygen shortage hindered the growth of *A. philoxeroides* (Figure 1), similar to the findings of previous studies [3,35,37]. Both stem and root growth were restricted by low dissolved oxygen levels (Figure 1 and Figure 2). The plants grown under low dissolved oxygen conditions produced a lower total number of roots with lower total length (Figure 3A,B). It is known that growth of plant tissues is an active, energy-dependent process [5]. Aerobic respiration is more efficient than anaerobic respiration and produces more ATP [4]. Both nutrient and water uptake by roots are regulated by ATP production [6]. Therefore, the reduced plant growth and root production observed here are consistent with the known dependence of root growth and resource uptake on oxygen-dependent energy metabolism, although ATP production, respiration, nutrient uptake, and water uptake were not directly measured. Consequently, this hypoxic restriction forces the plants into a severe systemic energy deficit, requiring an active functional response.

Environmental heterogeneity is a common phenomenon in the natural world, and it significantly influences plant behavior at various levels of complexity [38]. A previous study showed that in a nutrient-homogeneous soil environment, the development of first- and second-order lateral roots was similar at all depths; however, in a nutrient-heterogeneous soil environment, root development was poor when nutrients were scarce, but root proliferation was very strong in nutrient-rich soil layers [39]. Changes in root architectural traits are expected to promote resource uptake and growth [40,41]. Similarly, we propose that root architectural plasticity acts as a primary foraging strategy under oxygen heterogeneity. In the present study, under 5% air saturation, root traits were unevenly distributed among submerged nodes at varying distances from the water surface. Root number, root length, root surface area, root volume, and root fork number generally increased toward nodes closer to the water surface, whereas under 100% air saturation, these traits were more evenly distributed among nodes (Figure 4 and Figure 5). However, when under 100% air-saturation conditions, the traits of roots formed on all nodes were more similar; the differences in root traits between roots formed on nodes with different distances to water surface were much smaller than when plants were under 5% air-saturation conditions. Naturally, oxygen in atmosphere diffuses into any contacting water bodies as long as they are not oxygen-saturated by the atmosphere. In our experiment, atmospheric oxygen could diffuse into the 5% and 50% air-saturation solutions from the solution surface, potentially creating vertical oxygen heterogeneity, with relatively higher oxygen availability near the surface than in deeper layers. In contrast, in 100% air-saturation treatment, because the solution was already oxygen-saturated by air, contact of solution surface with air cannot further increase oxygen concentration in the solution, and the oxygen concentration along the solution profile would stay the same. The oxygen concentration difference along the solution profile in 5% and 50% air-saturation treatments caused by inward atmospheric oxygen diffusion consequently affected the spatial pattern of root formation in *A. philoxeroides* plants, leading to more roots being produced and larger root number, root length, root surface area, root volume, and root fork number on nodes nearer than on nodes further from the solution surface, as compared to 100% air-saturation treatment.

Crucially, based on these distinct allocation patterns, we can infer the underlying energy trade-offs driving this spatial shift. By sacrificing the costly construction of deep roots in anoxic, low-return zones, plants strategically reallocate their restricted energy reserves to superficial nodes to maximize oxygen capture. Different from the well-known relationships between plant performance and nutrient and water availability, our findings provide a new insight into the responses of plants to another abiotic factor—oxygen—and disclose how plants cope with underground hypoxic conditions by executing this energy-driven root architectural plasticity.

### 3.2. Internal Gas Transport Capacity and Spatial Rooting Pattern of Wetland Plants

Aerenchyma is a chief tissue containing large gas-filled spaces (lacunae) that interconnect longitudinally to provide a low-resistance pathway for long-distance gas transport along plant organs. It enhances internal aeration between and within shoots and roots. Aerenchyma formation is therefore important for the adaptation of plants in environments with excess water, such as waterlogged or submerged plants. In many wetland plants, aerenchyma forms constitutively and is further enhanced in response to flooding and submergence [9,10]. The roots of many wetland species contain aerenchyma as well as a barrier against radial O_2_ loss (ROL), which further enhances O_2_ movement to the apex [42,43,44] and supplies O_2_ to the deeper soil. In our present study, we found that hypoxic underground environments induced stronger root production in shallow substrate layers but weaker rooting in deep layers, and oxygen-rich substrate induced not only more roots but a more even distribution of roots along the substrate profile as well. Based on our results, it is clear that underground oxygenation status controlled both how many roots were likely to be produced and where the roots were to be produced. It can be speculated that compared to plants without aerenchyma in their stems, rhizomes, and roots, wetland plants with well-developed aerenchyma systems are able to transport more oxygen to the root apex, releasing it into deep soils and improving the oxygenation level there, which in turn induces higher root production in the deep soils.

### 3.3. Underground Oxygen Status and Its Effect on Plants’ Mechanical Stability in Environments Prone to Gusts or Floods

It has long been recognized that wind causes physical and destructive damage to plants [45,46]. Kort [47] noted that wind causes various types of damage, including uprooting and windthrow. It was predicted that wind speed will increase extensively in the future because of climate change [48]. A healthy plant can flex in the wind, which helps prevent breakage during gusts or strong winds. However, even robust trees are susceptible to wind injury. During strong or severe wind storms, full canopies of tall plants can act as sails in the wind. When wind speed is high, the gusty wind may cause an entire plant to uproot. Roots anchor plants in soil. In general, the wider, deeper, and stronger the root system is, the better a plant can withstand gusts and other mechanical forces, such as fast flood currents. Root and soil properties together affect the plant uprooting process [49]. In wetlands, plants are waterlogged during most of their growth period, and their root systems are always under anoxic or hypoxic conditions. According to our findings, the level of oxygen availability in underground environments affected root growth and rooting spatial pattern, resulting in more shallow root systems under hypoxic conditions. Therefore, in wetlands, some plants, especially those that lack well-developed internal aeration systems, may develop weaker and shallower root systems under hypoxic conditions. When gusty winds or strong floods occur, these shallow-rooted plants (especially tall ones), usually growing on soft muds or soils, are liable to be uprooted and be blown or flushed away. However, if the underground environments are well oxygenated, whether by oxygen release from underground parts of plants or by any other measures, the spatial root distribution along soil profile of plants will be improved and comparatively deeper root systems are developed, which strengthens the capacity of plants to withstand lateral forces like gusty winds and strong flood currents.

### 3.4. Ecological Implications of Spatial Root Plasticity and Study Limitations

**Figure 6:**
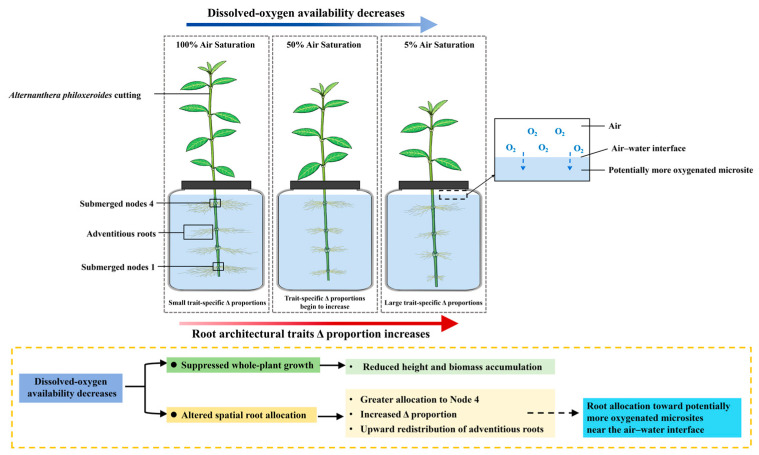
Conceptual model of dissolved oxygen-driven spatial rooting plasticity in *A. philoxeroides*. As dissolved oxygen availability decreases, *A. philoxeroides* shifts from uniform root development to an upward-concentrated distribution of adventitious roots, prioritizing biomass allocation toward the oxygen-rich air–water interface.

Using *A. philoxeroides* as a model shifts the focus from traditional internal anatomical and metabolic traits to a uniquely macroscopic ecological perspective [50]. By leveraging its extreme architecture plasticity, we successfully decouple general growth inhibition from active spatial foraging. Conceptually, our results support the view that dissolved oxygen availability may act as a spatially heterogeneous environmental factor influencing root system architecture [40]. To synthesize this interpretation, we present a conceptual model showing that declining dissolved oxygen availability may increase oxygen limitation in deeper solution layers, thereby favoring adventitious rooting at upper submerged stem nodes and leading to spatial redistribution of root growth toward the solution surface (Figure 6). This spatial rooting paradigm provides significant value for broader ecological applications. In highly fluctuating environments, such as reservoir drawdown zones, community assembly patterns, like the dominance of flood-tolerant perennials represented by *Cynodon dactylon* over annuals in high-stress, low-elevation areas, can be better understood through this lens [51]. The ability to dynamically adjust rooting depths in response to water levels ultimately dictates a plant’s mechanical stability and survival under prolonged flooding [52].

While our findings provide clear evidence of active spatial reconfiguration, we acknowledge certain constraints in our experimental approach. Although we did not perform micro-anatomical cross-sections, the observed increase in root surface-area-to-volume ratio (SA: V) under hypoxia strongly suggests a functional trade-off where plants prioritize oxygen acquisition over metabolic biomass investment. Similarly, while our allocation data are based on fresh weight, the use of a fully controlled hydroponic system where water availability was uniform across all oxygen treatments ensures that these relative patterns remain a robust proxy for spatial strategy. In addition, while the clonal identity of our cuttings was not genetically verified, the consistent, independent response observed across replicates grown in separate bottles supports the internal validity of our findings regarding oxygen-driven spatial plasticity. Finally, as the underlying biochemical drivers were not the primary focus here, future work integrating layer-specific metabolomic profiling with targeted anatomical staining will be essential to fully bridge the gap between macroscopic spatial escape and micro-physiological tolerance [53].

## 4. Materials and Methods

### 4.1. Plant Materials

**Figure 7:**
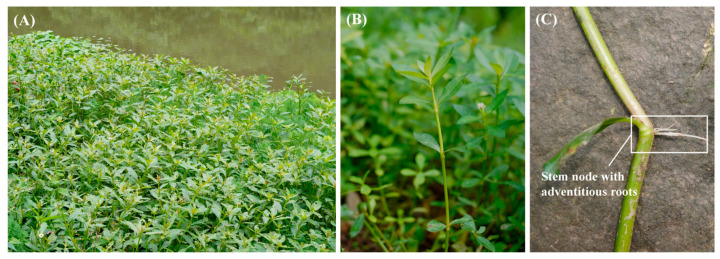
Representative photographs of *A. philoxeroides* in its natural habitat. (**A**) Natural population of *A. philoxeroides* growing along a riparian habitat. (**B**) Representative shoot architectural traits showing the slender stem and opposite leaves. (**C**) Close-up of a stem node bearing adventitious roots. These photographs are provided to illustrate the general architectural traits, growth habit, and habitat context of the study species.

*A*. *philoxeroides* was used as the experimental species in this study. *A. philoxeroides* is a perennial clonal plant in the family Amaranthaceae. It is native to South America but has now spread to many regions worldwide. This species commonly occurs in riparian zones, wetlands, and aquatic–terrestrial transition habitats, and shows high tolerance to flooding. Both the stems and rhizomes of *A. philoxeroides* bear distinct nodes. Under flooded conditions, submerged stem nodes can produce abundant aquatic adventitious roots [54]. Therefore, this species is suitable for investigating the effects of dissolved oxygen levels on adventitious root formation at submerged nodes and on the spatial distribution of roots. Representative photographs showing the natural habitat, shoot architectural traits, and adventitious root formation of *A. philoxeroides* are provided in Figure 7.

The plant materials used in this experiment were collected from multiple visually distinguishable ramets within a natural population of *A*. *philoxeroides* along the Jialing River in Chongqing, China (29°49′ N, 106°25′ E). During collection, plants approximately 25 cm in height, with good growth status, unbranched stems, intact leaves, and no visible disease, pest damage, or mechanical injury, were selected from the natural population. These plants were cut at the basal part of the stem to prepare cuttings. The collected cuttings were then transported to the laboratory, where cuttings with similar growth status, intact leaves, and no visible adventitious roots on the nodes were further selected for the subsequent experiment.

### 4.2. Experimental Design

Full-strength standard Hoagland solution was used to hydroponically culture the *A*. *philoxeroides* cuttings in this study. Three air-saturation treatments were established, corresponding to 100%, 50%, and 5% air saturation. A total of 30 cuttings were used, with 10 replicates assigned to each dissolved oxygen level. Each cutting was regarded as an independent experimental replicate and fixed in a plastic bottle.

The full-strength Hoagland solution was prepared with deionized water. Its nutrient composition was as follows (mmol L^−1^): 6.5 K^+^, 14.5 NO_3_^−^, 2 NH_4_^+^, 2 Mg^2+^, 4 Ca^2+^, 2 H_2_PO_4_^−^, 2 SO_4_^2−^, 0.0046 BO_3_^3−^, 0.0005 Mn^2+^, 0.045 Cl^−^, 0.0002 Zn^2+^, 0.0001 MoO_4_^2−^, 0.0002 Cu^2+^, and 0.045 Fe^3+^. The pH of the nutrient solution was adjusted to 6.5 using NaOH.

Before the treatments were initiated, the leaves on the lower four stem nodes that were to be submerged were removed, and the initial stem height and total fresh mass of each cutting were measured. No visible roots had formed on any of the cuttings before the treatments. Therefore, all roots measured at the end of the experiment were adventitious roots newly formed from the submerged stem nodes during the treatments.

Each cutting was placed individually in a 1000 mL plastic bottle containing Hoagland solution with the corresponding dissolved oxygen level. The lid of each bottle had two holes: one hole was used to hold the cutting upright, and the other was used to insert a tube for gas bubbling into the nutrient solution. The cuttings were positioned so that the lower four defoliated stem nodes were completely submerged in the nutrient solution. The lowermost submerged stem node of each cutting was defined as the 1st node, whereas the submerged node closest to the solution surface was defined as the 4th node. The lids were not sealed airtight; the holes allowed gas exchange between the bottle headspace and the ambient atmosphere, so the pressure inside the bottles remained approximately atmospheric.

Dissolved oxygen levels in the nutrient solutions were adjusted and maintained by bubbling air/N_2_ gas mixtures into the culture bottles. N_2_ was supplied from a high-pressure nitrogen cylinder and passed through a pressure regulator before entering the gas line, whereas air was supplied by an air pump. The two gas streams were mixed before entering the bottles, and the relative input of air and N_2_ was adjusted to achieve the target dissolved oxygen levels in the nutrient solutions. During the experiment, all bottles were gently bubbled with the corresponding air/N_2_ gas mixture twice daily for 120 min each time to maintain the assigned dissolved oxygen treatment levels. The nutrient solution for each cutting was renewed every two days until the end of the experiment. After each solution renewal, the corresponding air/N_2_ gas mixture was immediately bubbled into the bottle to re-establish the assigned dissolved oxygen level in the freshly replaced Hoagland solution.

**Figure 8:**
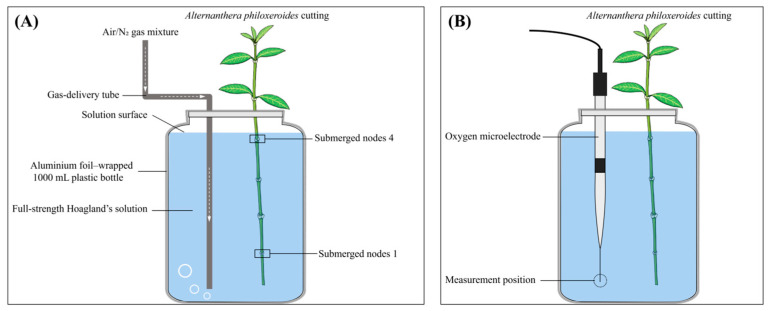
Schematic diagram of the hydroponic dissolved oxygen treatment system and oxygen partial pressure measurement in *A. philoxeroides*. (**A**) Hydroponic setup used for dissolved oxygen treatments. Each cutting was grown individually in a 1000 mL plastic bottle containing Hoagland nutrient solution, with four defoliated stem nodes submerged in the solution. Dissolved oxygen levels were adjusted by gently bubbling air/N_2_ gas mixtures into the nutrient solution through a gas inlet tube. The lowermost submerged node was defined as the 1st node, and the submerged node closest to the solution surface was defined as the 4th node. (**B**) Oxygen partial pressure was monitored using an oxygen microelectrode inserted into the middle position of the Hoagland nutrient solution to verify the assigned dissolved oxygen treatment level in each bottle. Diagrams are not drawn to scale.

Oxygen partial pressure in the nutrient solution was measured using an oxygen electrode with a 50 μm tip diameter (OX50, Unisense, Aarhus, Denmark). Measurements were taken at the middle position of the nutrient solution in each bottle. Oxygen partial pressure was measured twice daily, once in the morning and once in the evening, to monitor the maintenance of the dissolved oxygen treatments. The oxygen partial pressures corresponding to the 100%, 50%, and 5% air-saturation treatments during the experiment were 20.6 ± 0.102, 10.2 ± 0.038, and 1.04 ± 0.029 kPa, respectively. The pH of the nutrient solution was maintained at 6.5 ± 0.11 during the experiment (values are means ± SE). The hydroponic dissolved-oxygen treatment system and the position used for oxygen partial-pressure measurement are illustrated in Figure 8. Prior to the formal experiment, a preliminary trial was conducted to determine the aeration frequency required to maintain the target dissolved oxygen (DO) levels. During the preliminary trial, DO concentrations were measured twice daily (07:00 and 21:00 h) using a dissolved oxygen meter at two depths within each bottle: 3 cm below the water surface and 2 cm above the bottle bottom. Following each measurement, aeration was applied to restore the designated oxygen levels. Mean DO values recorded during the preliminary trial are presented in Table S1. Representative photographs of the hydroponic experimental setup and adventitious roots formed on submerged stem nodes are provided in Supplementary Figures S1 and S2, respectively.

The plastic bottles were wrapped with aluminum foil to keep the submerged stem segments and nutrient solution in darkness. The bottles were then placed in a GXZ-300D intelligent light incubator (Ningbo Dongnan Instruments Co., Ltd., Ningbo, China) under a 12 h light/12 h dark photoperiod. During the light period, photosynthetically active radiation at plant height was adjusted to approximately 280 μmol m^−2^ s^−1^ using the built-in illumination system of the incubator. This light level was used to provide sufficient illumination for the aerial leaves of the cuttings and to avoid light limitation during the experiment. The temperature was maintained at 25.0 °C during both the light and dark periods. All plants were randomly repositioned within the growth chamber once per day to minimize potential positional effects.

### 4.3. Plant Measurements

The experiment lasted for 23 days, after which all plants were harvested. Stem height, total fresh mass of each plant, and fresh mass of newly formed adventitious roots were measured. For each harvested plant, adventitious roots formed on the four submerged stem nodes were carefully separated by node before root analysis. Thus, roots produced on the 1st, 2nd, 3rd, and 4th submerged nodes were treated as separate node-specific samples for each individual plant.

Root number was recorded for each submerged node as the number of first-order adventitious roots emerging directly from that node. Before scanning, root samples from each node were gently rinsed with deionized water to remove residual nutrient solution and surface debris while avoiding damage to fine roots. Each node-specific root sample was then placed separately in a transparent scanning tray containing a thin layer of water. The roots were carefully spread with forceps to minimize overlap, crossing, and clumping, so that individual roots and branches were clearly visible in the scanned image. Each sample was scanned separately using a flatbed scanner and analyzed with WinRHIZO Pro 2004c (Regent Instruments Inc., Québec, QC, Canada). The same scanning and image-analysis settings were applied to all samples.

The scanned images were analyzed to obtain quantitative root morphometric and branching traits for each submerged node, including total root length, root surface area, root volume, and root fork number. Root fork number was used as an indicator of root branching complexity. These node-specific measurements allowed us to quantify not only overall root production, but also the spatial distribution of root traits along the submerged stem axis.

### 4.4. Data Analysis

The relative growth rates of fresh weight (RGR_FW_) and stem height (RGR_H_) were calculated for each plant using the following formula:

RGR (g∙g^−1^∙day^−1^ or cm∙cm^−1^∙day^−1^) = (lnA_2_ − lnA_1_)/(t_2_ − t_1_)

where A_1_ and A_2_ refer to the fresh weight or stem height of each plant at the start and end of the treatments, respectively, and t_1_ and t_2_ refer to the start and end times of the treatments, respectively.

To explore the difference in rooting intensity of underwater (underground) nodes with different distances to water surface, proportion of root number (or root length, root surface area, root volume, root fork number) on each node to those of all nodes was analyzed, and the proportion difference (Δ proportion) between the 4th node and 1st node (four nodes underwater in total each plant) in root number (or root length, root surface area, root volume, and root fork number) was investigated for each plant using the following formula:

Δ proportion of root number (or root length, root surface area, root volume, root fork number) = proportion of root number (or root length, root surface area, root volume, root fork number) growing on the 4th node-proportion of root number (or root length, root surface area, root volume, root fork number) growing on the 1st node, where the proportion of root number (or root length, root surface area, root volume, root fork number) was calculated as follows: root number (or root length, root surface area, root volume, root fork number) growing on the 4th node (or the 1st node)/the number of all roots formed on four submerged nodes during the treatments. A positive difference indicates that a larger proportion of the corresponding root trait was allocated to the node closest to the solution surface, whereas a negative difference indicates a larger proportion allocated to the lowermost submerged node.

For statistical analyses, each individual cutting grown in a separate culture bottle was treated as one independent biological replicate. Each dissolved oxygen treatment contained 10 independent plants, and the experiment included 30 plants in total. One-way ANOVA was conducted to investigate the effects of dissolved oxygen treatment on all parameters tested in the present study. Logarithm transformation of data was performed to equalize variance, if necessary. Differences between treatments were detected using Duncan’s multiple range test, and significant differences were reported at *p* < 0.05. All analyses were performed using SPSS 22.

## 5. Conclusions

Underground oxygen deficiency not only impedes the overall growth and root production of *A. philoxeroides*, but also fundamentally alters its macroscopic spatial rooting pattern. Severe hypoxic conditions (5% air saturation) significantly decreased plant relative growth rates, fresh mass allocation to roots, and total root length, while inducing the formation of more slender roots with a higher surface area-to-volume ratio (SA: V). Most importantly, under severe vertical oxygen limitations, plants actively concentrate their root development—exhibiting increased root number, length, surface area, and ramification—on the shallowest nodes near the atmosphere–water interface. Conversely, fully oxygenated environments remove this spatial constraint, allowing for robust root proliferation across all depths, including deeper substrate layers. Our results confirm that this spatial reallocation of roots toward surface layers serves as a primary, active architectural strategy for plants to manage systemic energy deficits. By decoupling overall growth inhibition from targeted spatial foraging, *A. philoxeroides* demonstrates a robust capacity for architectural plasticity, providing a critical ecological mechanism that ensures survival in fluctuating, hypoxic subterranean environments.

## Data Availability

Data are contained within the article and Supplementary Materials. Further inquiries can be directed to the corresponding authors.

## Supplementary Materials

The following supporting information can be downloaded at: https://www.mdpi.com/article/10.3390/plants15142137/s1. Figure S1: Photographs of the hydroponic dissolved oxygen treatment system used in this study; Figure S2: Representative photographs of adventitious root formation on submerged stem nodes of Alternanthera philoxeroides grown in Hoagland nutrient solution; Table S1: Mean dissolved oxygen saturation (%) at different depths under the three oxygen treatments during the preliminary experiment.
