# Supplementary material for: Underground Oxygen Deficiency Alters the Spatial Distribution Pattern of Rooting in Alternanthera philoxeroides
Source: Plants (Basel). 2026 Jul 10;15(14):2137. doi: 10.3390/plants15142137 (PMC13416283; doi:10.3390/plants15142137)
Supplement: Supplementary file 1 [file plants-15-02137-s001.zip › plants-4343742-supplementary.pdf]

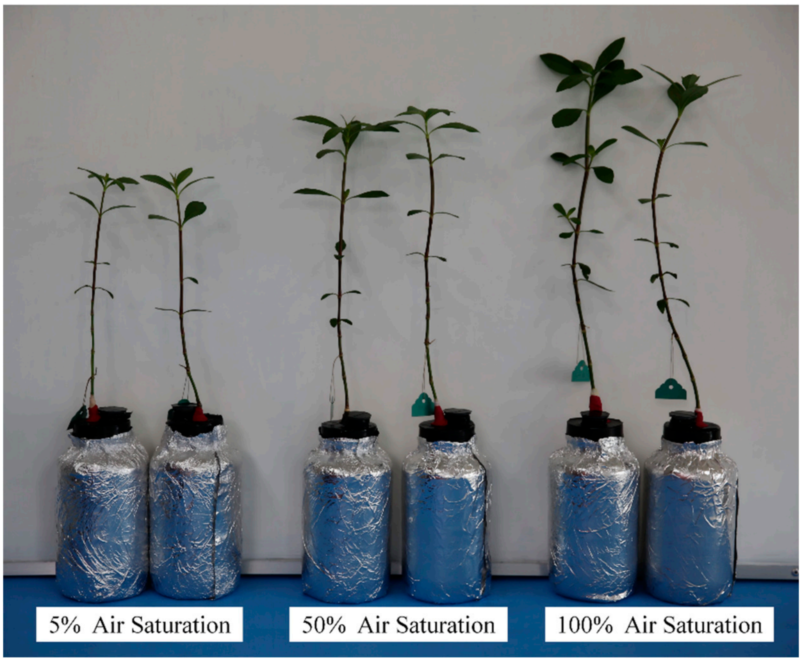

Figure S1. Photographs of the hydroponic dissolved oxygen treatment system used in this study. Individual cuttings of *Alternanthera philoxeroides* were grown in 1000-mL plastic bottles containing Hoagland nutrient solution. The bottles were wrapped with aluminum foil to exclude light from the submerged stem segments and nutrient solution. Four stem nodes were submerged in the solution, and dissolved oxygen levels were controlled by bubbling air/N_2 gas mixtures into the bottles throughout the experiment.

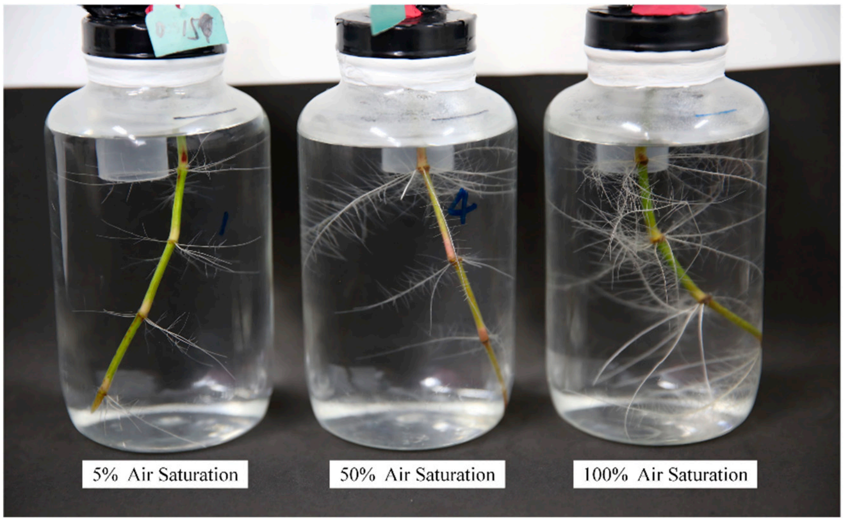

Figure S2. Representative photographs of adventitious root formation on submerged stem nodes of *Alternanthera philoxeroides* grown in Hoagland nutrient solution. Adventitious roots were produced from all submerged stem nodes during the experiment. Differences in root abundance and spatial distribution among submerged nodes can be visually observed.

Table S1. Mean dissolved oxygen saturation (%) at different depths under the three oxygen treatments during the preliminary experiment.

Treatments	Water temperature	DO saturation (%)	
		3cm below under water surface	2cm above bottom of bottle
5% air saturation	25.5±0.13℃	35.2±0.85%	12.1±0.35%
50% air saturation	25.4±0.14℃	70.6±0.38%	60.3±0.13%
100% air saturation	25.5±0.18℃	99.4±0.62%	99.8±0.28%

Values represent dissolved oxygen saturation measured immediately before scheduled aeration events during the preliminary experiment and are presented as mean ± SE.
